# The Sources of Inflammatory Mediators in the Lung after Silica Exposure

**DOI:** 10.1289/ehp.7295

**Published:** 2004-08-16

**Authors:** K. Murali Krishna Rao, Dale W. Porter, Terence Meighan, Vince Castranova

**Affiliations:** Pathology and Physiology Research Branch, Health Effects Laboratory Division, National Institute for Occupational Safety and Health, Morgantown, West Virginia, USA

**Keywords:** alveolar macrophages, alveolar type II cells, cytokines, fibroblasts, gene expression, lung, silica

## Abstract

The expression of 10 genes implicated in regulation of the inflammatory processes in the lung was studied after exposure of alveolar macrophages (AMs) to silica *in vitro* or *in vivo*. Exposure of AMs to silica *in vitro* up-regulated the messenger RNA (mRNA) levels of three genes [interleukin-6 (*IL-6*), monocyte chemoattractant protein-1 (*MCP-1*), and macrophage inflammatory protein-2 (*MIP-2*)] without a concomitant increase in the protein levels. AMs isolated after intratracheal instillation of silica up-regulated mRNA levels of four additional genes [granulocyte/macrophage-colony stimulating factor (*GM-CSF*), *IL-1*β, *IL-10*, and inducible nitric oxide synthase]. IL-6, MCP-1, and MIP-2 protein levels were elevated in bronchoalveolar lavage fluid. Fibroblasts under basal culture conditions express much higher levels of IL-6 and GM-CSF compared with AMs. Coculture of AMs and alveolar type II cells, or coculture of AMs and lung fibroblasts, in contact cultures or Transwell chambers, revealed no synergistic effect. Therefore, such interaction does not explain the effects seen *in vivo*. Identification of the intercellular communication *in vivo* is still unresolved. However, fibroblasts appear to be an important source of inflammatory mediators in the lung.

Occupational exposure to crystalline silica is associated with the development of pulmonary silicosis (Hnizdo and Vallyathan 2003; Reiser and Last 1979) and an increased risk for lung cancer (McDonald 1996). Silica can cause direct DNA damage and mammalian cell transformation (Daniel et al. 1995; Shi et al. 1994). The initial event, however, is an inflammatory response, including oxidant production and recruitment of inflammatory cells into the lung.

Numerous inflammatory mediators have been implicated in silica-induced pathology. Among them are cytokines, such as interleukin-(IL) 1β(Goodman et al. 1982), IL-6 (Gosset et al. 1991), IL-10 (Huaux et al. 1998), tumor necrosis factor-α(TNF-α) (Dubois et al. 1989), and transforming growth factor (TGF) (Williams et al. 1993; Williams and Saffiotti 1995); chemokines, such as monocyte chemoattractant protein-1 (MCP-1) (Barett et al. 1999) and macrophage inflammatory protein-2 (MIP-2) (Driscoll et al. 1993); the nonprotein inflammatory mediator nitric oxide, generated mainly through inducible nitric oxide synthase (iNOS) (Castranova et al. 1998); and adhesion molecules, such as intercellular adhesion molecule-1 (ICAM-1) (Hubbard and Giardina 2000; Nario and Hubbard 1996). However, the changes in the inflammatory mediators have been studied in different contexts. Some were studied *in vitro*, some *in vivo*, some in cell lines, and some in primary cells, making it difficult to conclude which of these mediators are involved in the initial phase of the lung inflammatory response and which become important in later stages of the response. A second aspect that has not received much attention is the source of these inflammatory mediators and the importance of cell–cell interactions in the production of these mediators.

In our studies of silica-induced production of inflammatory mediators in alveolar macrophages (AMs), we found the responses of AMs after *in vitro* stimulation were quite different compared with AMs isolated after *in vivo* exposure to silica. This suggests that cell–cell interactions may a play an important role in silica-induced production of inflammatory mediators in the lung. Previous studies have indicated a role for interaction between alveolar epithelial type II cells and AMs in the production of iNOS (Pechkovsky et al. 2002) and a role for interaction between fibroblasts and AMs in the production of granulocyte/macrophage-colony stimulating factor (GM-CSF) (Fitzgerald et al. 2003).

The recent technical advances in polymerase chain reaction (PCR) methodology make it possible to study the expression of several genes simultaneously even with small amounts of RNA. Therefore, to understand the role of inflammatory mediators in silica-induced pathology, we studied the expression of several inflammatory mediators in AMs after *in vitro* exposure to silica or after *in vivo* exposure by intratracheal instillation. The expression was studied at the message level by real-time reverse transcription (RT) PCR and, where appropriate, at the protein level by enzyme-linked immunosorbent assays (ELISAs). In addition, we studied the interactions between AMs and type II cells or fibroblasts in *in vitro* culture systems. Our studies indicate that the lung fibroblasts are an important source of inflammatory mediators after silica exposure.

## Materials and Methods

### Animals.

The animals used in these experiments were specific pathogen-free Sprague-Dawley rats [HLA:(SD)CVF; Hilltop Laboratories, Scottdale, PA] weighing 250–300 g (~ 8 weeks old at arrival). The animals were housed in an environmentally controlled facility that was accredited by the Association for Assessment and Accreditation of Laborary Animal Care. The rats were monitored to be free of endogenous viral pathogens, parasites, mycoplasmas, *Helicobacter*, and cilia-associated respiratory bacillus. Rats were acclimated for at least 5 days before use and were housed in ventilated cages, which were provided with HEPA-filtered air and used Alpha-Dri virgin cellulose chips (Shepherd Specialty Papers, Watertown, TN) and hardwood Beta chips (NEPCO, Warrensburg, NY) as bedding. The rats were maintained on 2018S Teklad Global 18% rodent diet (Harlan Teklad, Madison, WI) and tap water, both of which were provided ad libitum.

### Reagents.

Rat cytokine kits for IL-6, MCP-1, MIP-2, and TNF-αwere obtained from Biosource (Camarillo, CA). Lactate dehydrogenase (LDH) was measured within 24 hr on refrigerated samples with a COBAS MIRA Plus analyzer (Roche Diagnostics, Indianapolis, IN) using kits from Roche. Lipopolysaccharide B (LPS; from *E. coli* 026:B6) was obtained from Difco Laboratories (Detroit, MI). The culture medium consisted of Dulbecco’s modified Eagle medium (BioWhittaker, Walkersville, MD), 1 mM glutamine (Sigma, St. Louis, MO), 10 mM *N*-[2-hydroxyethyl]piperazine-*N*′-[2-ethane-sulfonic acid] (HEPES; Sigma), 100 U/mL penicillin–streptomycin (GIBCO Life Technologies, Grand Island, NY), 100 μg/mL kanamycin (GIBCO), and 10% (vol/vol) heat-inactivated fetal bovine serum (GIBCO).

### Source of silica.

We obtained MIN-U-SIL 5 from U.S. Silica (Berkeley Springs, WV). It was examined by proton-induced X-ray emission spectrometry for inorganic contaminants and for desorbable organic compounds by gas chromatography mass spectroscopy. The results of these analyses have been reported elsewhere (Porter et al. 2001). Silica samples were found to be > 99% pure quartz. Mean particle count diameter, determined by scanning electron microscopy, was 2.14 μm, with 99% of the particles < 5 μm. Silica was weighed and dry heated at 170°C for 24 hr to sterilize. Sterile medium was then added to the silica, which was vortexed into suspension before being added to the cell culture.

### Isolation of AMs.

The animals were anesthetized with pentobarbital sodium (150 mg/kg body weight) and exsanguinated by cutting the abdominal aorta. AMs were obtained by bronchoalveolar lavage (BAL) according to the method of Myrvik et al. (1961). The lungs from each animal were lavaged eight times with 5 mL phosphate-buffered medium (145 mM NaCl, 5 mM KCl, 9.4 mM Na_2_HPO_4_, and 1.9 mM NaH_2_PO_4_, pH 7.4) per gram lung weight. The cells were separated from the lavage fluid by centrifugation at 300 × *g* for 5 min and then washed three times by alternate centrifugation and resuspension in phosphate-buffered medium. The cells were then resuspended in the culture medium for use in all experiments. Cell number was determined by an electronic cell counter (model ZB; Coulter Electronics, Hialeah, FL).

### Isolation of type II cells.

We isolated type II cells as described previously (Miles et al. 1997). Briefly, the procedure involves perfusing the lung to remove blood, removing free AMs by BAL, digestion of lung tissue with elastase, and purification of type II cells by centrifugal elutriation. Cells isolated and purified by this method were > 85% pure type II cells as determined microscopically after staining with phosphine 3R (Jones et al. 1982).

The cells were cultured on collagen gels similar to those described by Lee et al. (1984) for growing hamster tracheal epithelial cells. Collagen gels were prepared from stock solution of collagen type I from rat tail (Sigma-Aldrich, St. Louis, MO) dissolved in 1:1,000 dilution of acetic acid in sterile distilled water overnight at 4°C. A six-well plate was layered with 0.775 mL (each well) of ice-cold collagen gel mixture consisting of 0.5 mL collagen stock, 0.15 mL 10× modified Eagle medium, and 0.125 mL 0.5 N NaOH. The mixture was allowed to polymerize for 4 hr at a humidified atmosphere of 5% CO_2_ at 37°C. The polymerized collagen gels were washed with 1 mL epithelial cell growth medium before cells were plated and grown overnight.

### Isolation of lung fibroblasts.

We isolated lung fibroblasts as described by Reist et al. (Reist et al. 1991). Briefly, the lungs were perfused with normal saline, lavaged with phosphate-buffered saline (PBS) containing 0.1% glucose, and sectioned four times at 0.5-mm intervals with a McIlwain tissue chopper. The chopped lung tissue from a single rat was digested in 20 mL of HEPES-buffered solution (145 mM NaCl, 5 mM KCl, 1 mM CaCl_2_, 505 mM glucose, and 10 mM HEPES, pH 7.4), containing collagenase (0.1%), elastase (40 U/mL), bovine serum albumin (0.5%), and DNAse (0.018%) in a shaker water bath for 30 min at 37°C. The digested mixture was filtered through two layers of sterile gauze that had been washed with culture medium. The cells were sedimented by centrifugation and plated in six-well culture plates. The medium was changed 24 hr later, and the cells were allowed to grow to confluence.

### Coculture of type II cells and AMs.

Type II cells cultured overnight on collagen gels in six-well plates (catalog no. 353046; tissue culture treated by vacuum gas plasma, polystyrene, nonpyrogenic; Becton Dickinson, Franklin Lakes, NJ), as described above, were incubated for an additional 4 hr at 37°C in a CO_2_ incubator with freshly isolated AMs (1 million cells) with or without silica. Controls were type II cells alone with or without silica. The collagen gels were dissolved in a solution containing 1 mg of Sigma blend collagenase type F made up in 1 mL of type II cell growth medium for each well to be dissolved. The cells were then spun down and used for isolation of total RNA.

### Coculture of lung fibroblasts and AMs.

Lung fibroblasts were cultured until they became confluent. The cells were trypsinized and 2 × 10^6^ cells were plated in six-well plates. After overnight culture, freshly isolated AMs (2 × 10^6^ cells) were added to the wells and cultured for an additional 4 hr with or without silica. Controls were fibroblasts alone with or without silica. The culture medium was aspirated and spun down, and the supernatant was stored at –80°C. The cells were scraped and combined with the cell pellet from the above step and used for isolation of total RNA.

### Transwell experiments with fibroblasts and AMs.

To measure messenger RNA (mRNA) expression in separated cell populations and to study the interaction of soluble mediators released by cell populations on each other, we conducted experiments in Transwell chambers (CoStar, Corning, NY). For these experiments, cultured lung fibroblasts were trypsinized, and 1 million cells were plated in the outer well of a Transwell plate and cultured for an additional 24 hr. At the end of the 24-hr period, freshly isolated AMs (1 million cells) were placed in the inserts. Silica was added either to the macrophages in the inner wells or to the fibroblasts in the outer well and incubated for 4 hr. Total RNA was isolated from each population separately.

### Preparation of AM- and polymorpho-nuclear neutrophil–enriched fractions.

We obtained AM- and polymorphonuclear neutrophil (PMN)–enriched fractions from BAL fluid obtained from rats treated with silica *in vivo*, as described by Huffman et al. (2003). Briefly, the method consisted of layering BAL cell populations obtained by lavage onto a Histopaque double-density gradient composed of equal amounts of Histopaque 1083 and Histopaque 1119 (Sigma). The gradients were then centrifuged (400 × *g*, 30 min, room temperature). The AM-enriched fraction localized at the interface between PBS diluent and Histopaque 1083, and the PMN-enriched fraction was located at the bottom as a pellet. This method yields about 60% AMs in the AM-enriched fraction and 90% PMN in the PMN-enriched fraction (Huffman et al. 2003).

### Measurement of cytokines.

We measured the cytokines in culture supernatants after a 24-hr incubation with either 200 μg/mL silica or 1 μg/mL LPS. IL-6, MCP-1, MIP-2, and TNF-α were measured by ELISA kits according to manufacturer instructions (Biosource International, Camarillo, CA). The values were expressed as nanograms or picograms per million cells. For measurement of cytokines in the BAL fluid, lavage fluid from the first wash was collected and spun down to sediment the cellular elements. The supernatant was stored at –80°C for later measurement of cytokine levels by ELISA.

### Quantitation of mRNAs by RT-PCR.

We measured cytokine mRNA levels using a SYBR Green PCR kit with the ABI 5700 Sequence Detector (PE Applied Biosystems, Foster City, CA). Total RNA was isolated using RNAqueous 4PCR kits (Ambion, Austin, TX) from AMs (≈2 million cells) or lung tissue after alveolar lavage (≈50 mg wet tissue). One to two micrograms of the DNAse I–treated RNA was reverse transcribed, using Superscript II (Life Technologies, Gaithersburg, MD). The complementary DNA generated was diluted 1:100, and 15 μL was used to conduct the PCR reaction according to the SYBR Green PCR kit instructions. The comparative *^C^T* (threshold cycle) method was used to calculate the relative concentrations (User Bulletin no. 2; ABI PRISM 7700 Sequence Detector, PE Applied Biosystems). Briefly, the method involves obtaining the *C_T_* values for the cytokine of interest, normalizing to a housekeeping gene (*18S* in the present case), and deriving the fold increase compared with the control, unstimulated cells. Table 1 lists the primer sets used for these experiments. In preliminary experiments, the products were analyzed by gel electrophoresis, and a single product was obtained with each primer set. In addition, dissociation curves yielded single peaks.

### In vitro *experiments.*

All experiments were performed on pooled AMs from several animals. AMs were placed in six-well plates, incubated for 2 hr at 37°C, and washed to remove nonadherent cells. Then the cells were incubated with silica (200 μg/mL) or LPS (1 μg/mL) for 4 hr for mRNA measurements, or 24 hr for the measurement of inflammatory cytokines.

### In vivo *experiments.*

Rats were anesthetized with an intraperitoneal injection of 30–40 mg/kg body weight sodium methohexital (Brevital; Eli Lilly and Company, Indianapolis, IN) and were intratracheally instilled using a 20-gauge 4-inch ball-tipped animal feeding needle. Silica (MIN-U-SIL 5) was suspended in endotoxin-free, Ca^2+^/Mg^2+^-free PBS (BioWhittaker, Walkersville, MD), and rats received either 2 mg silica/100 g body weight or an equivalent volume of PBS. The animals were sacrificed 4 hr postexposure, and AMs were isolated as described above. The lavaged lung tissue was used for isolation of total RNA.

### Statistical methods.

A paired *t*-test was used for *in vitro* experiments. A *t*-test assuming unequal variance or a *Z*-test for means was used to evaluate the *in vivo* data. The significance was set at < 0.05.

## Results

### Effects of silica treatment on cell viability.

In initial experiments, the effect of silica treatment (200 μg/mL) on cell viability was assessed by measuring the release of LDH into the medium at the end of the 4-hr incubation time. The means ± SEs (U/L) for control versus silica-treated cells (*n* = 3) were, for fibroblasts, 49 ± 14 versus 49 ± 15; for type II cells, 87 ± 9 versus 91 ± 7; and for macrophages, 101 ± 13 versus 100 ± 7.

### *Effects of silica or LPS on mRNA expression in AMs* in vitro.

**Figure 1:**
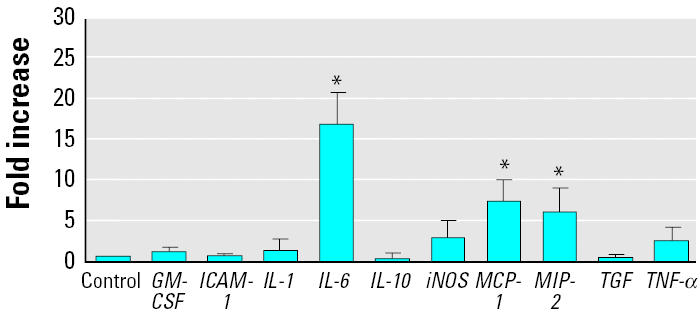
mRNA expression in AMs stimulated *in vitro* with silica (200 μg/mL) at 4 hr postexposure. Error bars represent fold increase above control (mean ± SE of at least four experiments for each cytokine).
*Significantly greater than control,
*p* < 0.05.

AMs were stimulated with either 200 μg/mL silica or 1 μg/mL LPS for 4 hr. The expression of 10 genes, implicated in the induction of an inflammatory response, was measured by real-time RT-PCR. The message levels of only three cytokines (*MCP-1*, *MIP-2*, and *IL-6*) showed a significant increase at 4 hr after *in vitro* exposure to silica (Figure 1). In contrast, mRNA levels for *GM-CSF*, *ICAM-1*, *IL-1*β, *IL-10*, *iNOS*, *TGF*-β*1*, and *TNF-*αwere not significantly elevated after this treatment.

**Figure 2:**
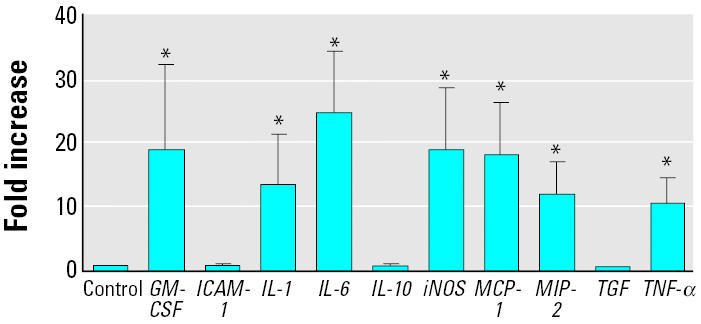
mRNA expression in AMs stimulated *in vitro* with LPS (1 μg/mL) at 4 hr postexposure. Error bars represent fold increase above control (mean ± SE) in the message levels from a minimum of four different experiments with LPS.
*Significantly different from control except *ICAM-1*, *IL-10*, and *TGF*-β*1* (*TGF*).

To compare the effect of silica with that of bacterial endotoxin, LPS, we also measured mRNA levels of these inflammatory mediators in AMs stimulated with LPS for 4 hr *in vitro* (Figure 2). LPS stimulation increased message levels of *IL-1*β, *IL-6*, *GM-CSF*, *iNOS*, *MCP-1*, *MIP-2*, and *TNF*-αbut not those of *ICAM-1*, *IL-10*, and *TGF-*β*1*.

### *Effects of* in vivo *silica treatment on mRNA expression in cells obtained by BAL.*

**Figure 3:**
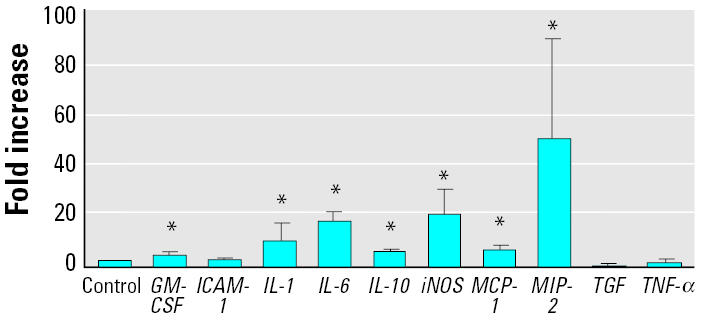
mRNA expression in cells obtained by BAL from animals at 4 hr after intratracheal instillation of silica (2 mg/100 g body weight). Error bars represent fold increase above control (mean ± SE) in the message levels from a minimum of five different animals (in the control and treated groups).
*Significantly greater than control,
*p* < 0.05.

Figure 3 shows the mRNA expression in cells isolated from rats 4 hr after intratracheal instillation of silica (2 mg/100 g body weight). The three cytokines that showed an increase at 4 hr *in vitro* (*IL-6*, *MCP-1*, and *MIP-2*) also showed an increase *in vivo*. In addition, the expression of four other genes (*GM-CSF*, *IL-1*β, *IL-10*, and *iNOS*) was increased. Three genes (*TGF-*β*1*, *TNF-*α, and *ICAM-1*) showed no change either *in vitro* or *in vivo*.

### Effects of silica on mRNA expression in the lung tissue after intratracheal instillation.

**Figure 4:**
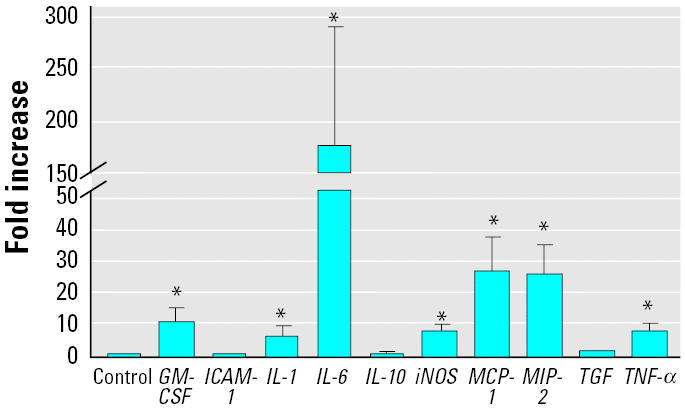
mRNA expression in the lavaged lung tissue isolated from animals at 4 hr after intratracheal instillation of silica (2 mg/100 g body weight). Error bars represent fold increase above control (mean ± SE) in the message levels from a minimum of five different animals (in the control and treated groups).
*Significantly greater than control,

We also measured cytokine expression in the lavaged lung tissue 4 hr after the intratracheal instillation of silica (Figure 4). The results were mostly similar to those seen in AMs (Figure 3). There was a significant increase in the message levels of *GM-CSF*, *IL-1*β, *IL-6*, *iNOS*, *MCP-1*, *MIP-2*, and *TNF-*α. No increase was seen for *ICAM-1*, *IL-10*, and *TGF-*β1.

### mRNA expression of cytokines in AM-enriched and PMN-enriched fractions.

**Figure 5:**
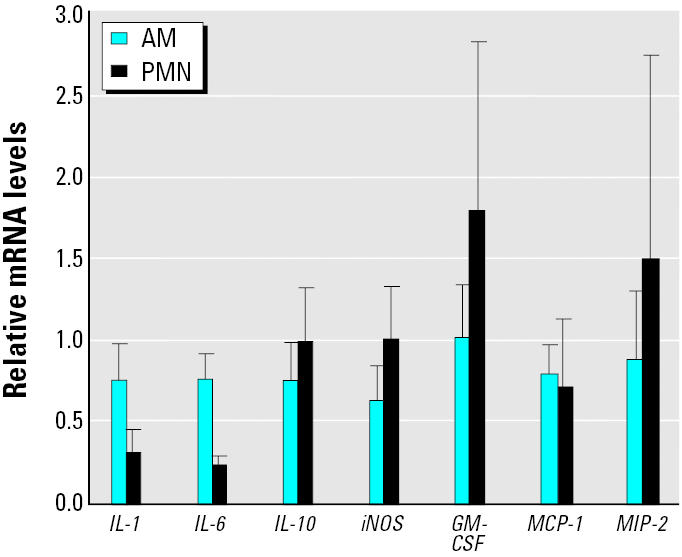
mRNA expression in separated AMs and PMNs isolated from animals at 4 hr after intratracheal instillation of silica (2 mg/100 g body weight). Error bars represent fold increase above control (mean ± SE) in the message levels from three different animals.

One major difference between cells obtained by BAL from control rats versus silica-treated rats is the presence of a large number of PMNs in the silica-treated animals. One explanation for the differences seen in gene expression after *in vitro* and *in vivo* exposure may be that the neutrophils produce additional cytokines not seen with AMs alone. To determine the role of PMN in mRNA expression after silica treatment, we obtained AM-enriched and PMN-enriched fractions from BAL fluid of animals treated with silica *in vivo*. The mRNA levels were expressed in relation to the expression levels in relation to AMs. These AMs have been exposed to silica *in vivo* and express high levels of mRNA, as shown in Figure 3. The mRNA expression in AMs was assigned an arbitrary value of 1 for Figure 5. Figure 5 shows that mRNA expression of the seven cytokines studied was essentially the same in the two fractions, indicating that PMN enrichment is not the cause for differences between *in vitro* and *in vivo* treatments.

### Cytokine/chemokine expression at the protein level.

**Figure 6:**
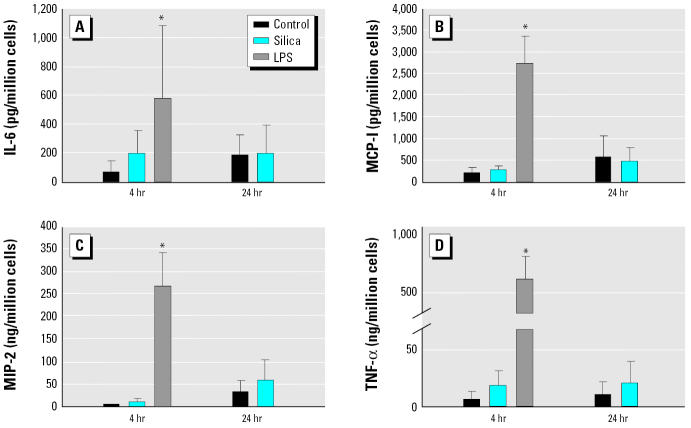
Cytokine/chemokine protein levels as determined by ELISA in culture supernatants of AMs treated *in vitro* with silica (200 μg/mL) or LPS (1 μg/mL) for 4 hr, or silica for 24 hr. There were no differences in (*A*) IL-6, (*B*) MCP-1, (*C*) MIP-2, or (*D*) TNF-αlevels between control and silica-treated cells at either exposure time. In contrast, LPS produced a large increase in all four cytokine/chemokines as early as 4 hr. Error bars represent mean ± SE from four separate experiments.
*Significantly greater than control,
*p* < 0.05.

**Figure 7:**
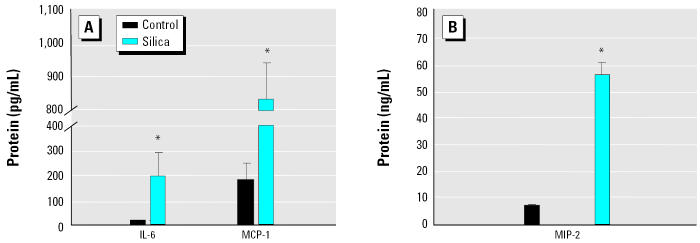
Cytokine/chemokine protein levels as determined by ELISA in alveolar lavage fluid from animals at 4 hr after the intratracheal instillation of silica (2 mg/100 g body weight). Error bars represent mean ± SE from five different animals (in the control and silica-treated groups). Silica treatment increased protein levels of the three mediators, (*A*) IL-6 and MCP-1 and (*B*) MIP-2.
*Significantly greater than control,
*p* < 0.05.

We measured the levels of four cytokines/chemokines (IL-6, MCP-1, MIP-2, and TNF-α) in the supernatants of AM cultures after 4 hr incubation with either silica or LPS. There was no increase in the protein levels of these mediators with silica, but LPS produced very high levels of these cytokines/ chemokines (Figure 6A–D). There was no increase in these mediators even after 24 hr incubation with silica. In contrast, the cytokine levels of IL-6, MCP-1, and MIP-2 were increased in the alveolar lavage fluid when the animals were exposed to silica for 4 hr *in vivo* (Figure 7). There was no increase in TNF-α levels 4 hr after exposure to silica either *in vitro* or *in vivo* (data not shown).

### Coculture of type II cells and AMs on gene expression.

To determine whether the differences seen in mRNA expression in AMs exposed to silica *in vitro* and *in vivo* may be related interaction between AMs and type II cells, we performed coculture experiments. We focused on four genes that were up-regulated only after *in vivo* exposure. Table 2 shows the expression of these four genes in cocultures of AMs and type II cells. Essentially, there was no difference in the expression of these genes when the cells were cocultured with or without silica. These results indicate that the expression of these inflammatory mediators is not mediated by interaction between AMs and type II cells.

### Gene expression in lung fibroblasts.

**Figure 8:**
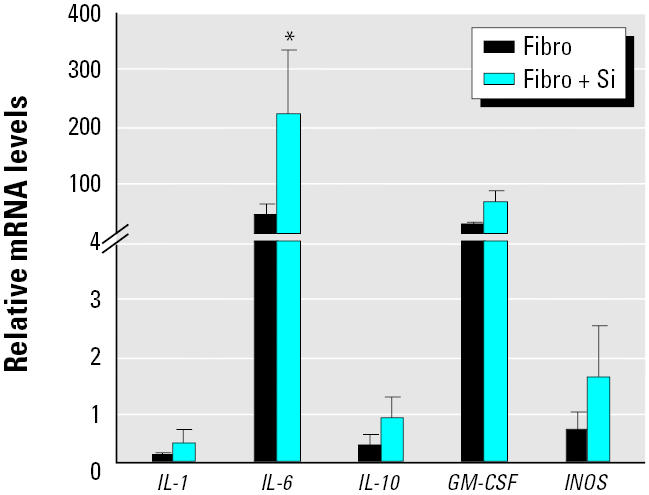
mRNA expression in lung fibroblasts (Fibro) stimulated *in vitro* with silica (Si; 200 μg/mL) at 4 hr postexposure. The mRNA values were measured relative to that found in freshly isolated AMs incubated for 4 hr. Error bars represent fold increase above control (mean ± SE of at least four experiments for each inflammatory mediator).
*Significantly greater than control,
*p* < 0.05.

Figure 8 shows the expression of five genes in fibroblasts cultured alone or in the presence of silica. We included *IL-6* because lung tissue showed very high levels of *IL-6* mRNA levels (Figure 4). The mRNA levels were expressed relative to mRNA levels of AMs alone. It is clear that the mRNA levels for *IL-6* and *GM-CSF* are very high in resting lung fibroblasts. IL-6 protein levels, as determined by ELISA, were 100-fold higher in culture supernatants of lung fibroblasts compared with culture supernatants of AMs (210 ± 75 vs. 2.4 ± 1.1, *n* = 7), indicating that the message is being translated into protein. In addition, *in vitro* exposure to silica caused a significant increase in mRNA levels, but this increase in mRNA levels was not reflected in an increase in protein synthesis (210 ± 75 vs. 231 ± 62, control vs. silica, *n* = 7). This is similar to that seen in AMs. We did not measure the GM-CSF protein levels.

### Gene expression in AMs and fibroblast cocultures.

Table 3 shows the relative expression of the five genes in coculture experiments. Although coculture of AMs and lung fibroblasts seems to enhance *iNOS* and *IL-10* mRNA levels seen over and above that seen with each cell type alone, the results were too variable to draw a definitive conclusion concerning a synergistic effect. With regard to *IL-6* and *GM-CSF*, the main source seems to be fibroblasts, but results were too variable to conclude whether cocultures with or without silica enhance the mRNA levels.

### mRNA expression in AMs and lung fibro-blasts in Transwell experiments.

The coculture experiments do not allow determination of mRNA expression in individual cell types. Therefore, we conducted Transwell experiments to isolate RNA from each cell type and to study the roles of cell–cell contact versus soluble mediators in these interactions (Table 4). Two conclusions can be drawn from these experiments: First, there is no difference in the mRNA levels of *IL-1*, *IL-10*, and *iNOS* under the different conditions tested; and second, the main sources of IL-6 and GM-CSF are lung fibroblasts. Although AMs seem to enhance *IL-6* and *GM-CSF* mRNA levels in fibroblasts, the extreme variation in the results does not permit a definitive conclusion.

Combining the observations from coculture experiments and Transwell experiments, it appears that factors in addition to cell–cell contact and soluble mediators secreted by these two cell types are involved in regulating the inflammatory mediators in *in vivo* situations.

## Discussion

Exposure to silica causes inflammatory and fibrotic lung disease (Hnizdo and Vallyathan 2003). Silica-induced inflammatory response has been implicated in the pathogenesis of fibrosis. In this study, we measured expression of 10 genes that are involved in regulating the inflammatory processes in the lung, at the message level. The studies were conducted in BAL cells and lung tissue after *in vivo* exposure, and in AMs, type II cells, and lung fibroblasts after *in vitro* exposure.

Exposure of AMs to silica *in vitro* increased message levels of only three genes: *IL-6*, *MCP-1*, and *MIP-2*. MCP-1 plays an important role in accumulation of monocytes (Leonard and Yoshimura 1990). MIP-2 is a potent chemotactic factor for neutrophils (Driscoll 1994). Up-regulation of these two genes very early, after silica exposure, may account for the rapid accumulation of these cells in the lung. IL-6 is a pleiotropic cytokine with multiple biologic activities (Van Snick 1990) that has been shown to be up-regulated after silica (Hetland et al. 2001) and asbestos (Simeonova et al. 1997) exposure of human lung epithelial cells. Here, we show that it is one of the early genes expressed in AMs after silica exposure. Up-regulation of these genes requires only the interaction between the silica particles and the macrophages.

When mRNA levels were measured in BAL cells harvested from rats instilled with silica, the mRNA levels of four other genes (*GM-CSF*, *IL-1*, *IL-10*, and *iNOS*) went up in addition to the three genes mentioned above.

The production of NO in isolated AMs from *in vivo* silica-treated animals and lack of NO production after *in vitro* treatment has been reported previously (Huffman et al. 1998). We confirm that observation. To determine whether cell–cell interactions may be involved in the production of NO and the expression of three other genes belonging to this group, AMs were cocultured with either type II cells or lung fibroblasts. The data (Table 2) clearly indicate that coculture with type II cells does not up-regulate these genes under any of the conditions studied.

Coculture of AMs with fibroblasts showed that *iNOS* and *IL-10* mRNA levels may go up, but the response was extremely variable, and no definitive conclusions could be drawn. In addition, the Transwell experiments show that the coculture of AMs and fibroblasts does not significantly increase the message levels of *GM-CSF*, *IL-1*, *IL-10*, and *iNOS* levels in AMs. Therefore, the factors responsible for up-regulation of these genes after intratracheal instillation of silica remains elusive.

Three other genes (*ICAM-1*, *TGF-1*β, and *TNF-*α) did not show any change in BAL cells and lung tissue after *in vivo* treatment or in AMs after *in vitro* treatment. The observation that the release of TNF-αis increased in the blood monocytes of miners with coal workers’ pneumoconiosis (Borm et al. 1988) has led to several studies showing an increase in TNF-α levels from AMs stimulated with silica (Baer et al. 1998; Dubois et al. 1989; Gossart et al. 1996). However, others have shown that *in vitro* treatment with silica does not induce TNF-αlevels in human AMs (Gosset et al. 1991). In some cases, where an increase in TNF-αproduction was shown, the levels were minimally increased and the levels were at least a couple of orders of magnitude less than what is seen with LPS stimulation (Kanj et al. 2002; Rojanasakul et al. 1999; Shi et al. 1999), raising the question of their biologic relevance. In one *in vivo* study in rats with silica, an increase in mRNA levels of *TNF-*αin AMs was not seen until 3 days after intratracheal instillation (Snadrin et al. 1996), and even later in a silica inhalation study (Porter et al. 2002). These data make the role of TNF-αin the initial stages of silica-induced inflammation questionable, even though TNF-αhas been reported to be a key mediator in the eventual development of fibrosis (Piguet et al. 1990). In our studies, we did not observe any increase in mRNA in AMs for *TNF-*αat 4 hr *in vitro* or at 4 hr *in vivo*. However, there was an increase in the mRNA expression in *TNF-*αin the lung tissue 4 hr after intratracheal instillation of silica.

The cytokine TGF-β1 and the adhesion molecule ICAM-1 have also been implicated in the pathogenesis associated with silica exposure (Matrat et al. 1998; Nario and Hubbard 1996). We have not detected any increase in the message levels of these two genes after either *in vitro* or *in vivo* silica exposure. TGF-β1 is shown to be critical in acute lung injury (Pittet et al. 2001) but may not play a role in particle-induced lung disease, at least in the initial stages. ICAM-1 has been shown to be up-regulated in LPS-induced lung inflammation (Madjdpour et al. 2000; Nathms et al. 1998). LPS stimulation *in vivo* has been shown to increase ICAM-1 expression both in AMs (Grigg et al. 1994) and in the lung tissue (Nathms et al. 1998). There was no increase in *ICAM-1* message in AMs after *in vitro* exposure to silica or LPS in the present study. A third gene that did not show any change with LPS was *IL-10*. Our findings are consistent with previous findings that there is no up-regulation of *TGF-*β*1* in AMs (Xing et al. 1994) or *IL-10* in lung tissue (Johnston et al. 1998) after LPS stimulation. With regard to *ICAM-1*, an increase was demonstrated in AMs after *in vivo* exposure to LPS (Grigg et al. 1994). We evaluated *ICAM-1* in AMs after only *in vitro* exposure.

We observed significant increases in the mRNA levels of *IL-6* and genes for two chemokines (*MCP-1* and *MIP-2*) at 4 hr after *in vitro* treatment with silica. Although the message levels showed an increase, there was no increase in the protein levels measured in the supernatants of the cultures at 4 hr. However, when silica was administered intra-tracheally, there was considerable increase in both message levels and protein levels at 4 hr. Our findings with regard to the production of MCP-1 and MIP-2 are consistent with previous observations demonstrating an increase in these two chemokines after silica exposure (Driscoll 2000; Driscoll et al. 1998; Hubbard et al. 2002). The observation that the *in vitro* treatment up-regulates the message levels without increasing the protein levels, but *in vivo* both message levels and protein levels go up, indicates that cell–cell interactions and/or other influences might play an important role in the expression of these cytokines at the protein level.

The Transwell experiments revealed that a major source of IL-6 and GM-CSF in the lung could be lung fibroblasts. When mRNA levels were expressed relative to AMs (Table 4), the *IL-6* levels in lung fibroblasts were several hundred-fold higher than those in AMs. Similarly, mRNA levels of *GM-CSF* were much higher in fibroblasts compared with AMs. Further, the number of fibroblasts (interstitial cells) is 10-fold higher than AMs in the lung tissue (Stone et al. 1992). These observations indicate that the fibroblasts are a major of source of these inflammatory mediators in the lung.

MCP-1 has significant involvement in the inflammatory disorders of the lung (Rose et al. 2003). It has been shown to regulate alveolar epithelial cell inhibition of fibroblast proliferation (Moore et al. 2002). In addition to monocytes, fibroblasts are an important source of MCP-1 (Galindo et al. 2001; Hao et al. 2003). We found *MCP-1* mRNA levels were severalfold higher in lung fibroblasts compared with AMs (data not shown). Therefore, the main source of both IL-6 and MCP-1 in the BAL fluid after silica exposure could be lung fibroblasts. This is consistent with the observation that silica can directly stimulate lung fibroblasts (Arcangeli et al. 2001; Baroni et al. 2001). We have not evaluated the sources of MIP-2 in this study.

GM-CSF is purported to play an important role in numerous respiratory illnesses, including asthma (Xing et al. 1996). It is generated by a variety of lung cell types (Bergman et al. 2000; Blau et al. 1994; Christensen et al. 2001; Churchill et al. 1992; Fitzgerald et al. 2003; O’Brien et al. 1998; Smith et al. 1990; Soloperto et al. 1991; Trapnell and Whitsett 2002). GM-CSF was not produced by AMs when stimulated with silica *in vitro*, but an increase in message levels were seen in both BAL cells and lung tissue after intratracheal instillation. This confirms the reported need for cell–cell interactions in the up-regulation of GM-CSF (Fitzgerald et al. 2003).

The importance of cell–cell interactions in the production of inflammatory mediators has been emphasized in several studies. Direct contact between human peripheral blood mononuclear cells and renal fibroblasts facilitates the expression of MCP-1 (Hao et al. 2003). Similarly, macrophage/fibroblast interactions are important for the production of GM-CSF (Fitzgerald et al. 2003). Both soluble mediators and adhesion molecules have been implicated in these interactions (Hao et al. 2003; Zickus et al. 2004). The lack of effect on the expression of several genes in coculture experiments with contact or without contact (Transwell experiments) indicates that some additional factors may be involved in the regulation of cytokine production in the lung after silica exposure. During inflammation a variety of cells are recruited into the lung and a number products are generated. Any one of these factors may influence the expression of inflammatory mediators. In this regard, it is important to keep in mind the role of lung surfactant. Lung surfactant is known to modulate immune functions in the lung (Wright 1997); we mention its role in particular because we have some preliminary data to suggest that lung surfactant may enhance cytokine production in the lung fibroblasts.

In summary, we found that exposure of AMs to silica *in vitro* up-regulates only three genes (*IL-6*, *MCP-1*, and *MIP-2*). However, in BAL cells harvested after intratracheal instillation of silica, four additional genes (*IL-1*, *IL-10*, *iNOS*, and *GM-CSF*) were up-regulated. Cocultures of AMs with alveolar epithelial type II cells or lung fibroblasts did not enhance mRNA level of the four additional genes that were expressed after *in vivo* exposure. There is need to evaluate the role of other mediators in regulating the production of inflammatory mediators in the lung, perhaps the role of lung surfactant. Most of the studies concerning silica-induced inflammatory processes in the lung have been focused on the role of AMs; our Transwell studies show that lung fibroblasts are an important source of IL-6 and GM-CSF. These observations indicate that the fibroblast-derived inflammatory mediators may also play an important role after silica exposure.

## Figures and Tables

*p* < 0.05.

**Table 1:** Primer sets used.

Gene	Primers	Product (bp)
*GM-CSF*	Sense: GAC ATG CGT GCT CTG GAG AAC G	144
	Antisense: GCC ATT GAG TTT GGT GAG GTT GC	
*ICAM-1*	Sense: AAT CTG ACC TGC AGC CGG AAA G	108
	Antisense: GGA GCT AAA GGC ACG GCA CTT G	
*IL-1*β	Sense: AGC TCC ACG GGC AAG ACA TAG G	155
	Antisense: GGA TGG CTT CCA AGC CCT TGA C	
*IL-6*	Sense: CCC AAC TTC CAA TGC TCT CCT AAT G	141
	Antisense: GCA CAC TAG GTT TGC CGA GTA GAC C	
*IL-10*	Sense: GGC TCA GCA CTG CTA TGT TGC C	116
	Antisense: AGC ATG TGG GTC TGG CTG ACT G	
*iNOS*	Sense: GTC ACC TAT CGC ACC CGA GAT G	117
	Antisense: GCC ACT GAC ACT CCG CAC AAA G	
*MCP-1*	Sense: TCA CGC TTC TGG GCC TGT TG	131
	Antisense: CAG CCG ACT CAT TGG GAT CAT C	
*MIP-2*	Sense: GGC AAG GCT AAC TGA CCT GGA AAG	113
	Antisense: CAC ATC AGG TAC GAT CCA GGC TTC	
*TGF-*β *1*	Sense: GCT AAT GGT GGA CCG CAA CAA C	103
	Antisense: TGG CAC TGC TTC CCG AAT GTC	
*TNF-*α	Sense: CGT CAG CCG ATT TGC CAT TTC	116
	Antisense: TGG GCT CAT ACC AGG GCT TGA G	
*18S rRNA*	Sense: GGA CCA GAG CGA AAG CAT TTG C	115
	Antisense: CGC CAG TCG GCA TCG TTT ATG	

**Table 2:** Relative mRNA expression in AMs and type II alveolar epithelial cell cocultures stimulated with silica (Si; 200 μg/mL) for 4 hr.

*mRNA*	AM	AM + Si	Type II	Type II + Si	AM + type II	AM + type II + Si
*IL-1*β	1.47 ± 0.53	0.87 ± 0.34	0.61 ± 0.41	0.41 ± 0.27	1.10 ± 0.60	1.03 ± 0.49
*IL-10*	1.04 ± 0.13	0.30 ± 0.11	2.58 ± 1.01	5.74 ± 2.62	1.41 ± 1.46	2.21 ± 2.32
*iNOS*	1.04 ± 0.12	0.67 ± 0.24	0.49 ± 0.23	0.44 ± 0.16	1.75 ± 1.63	1.93 ± 1.47
*GM-CSF*	1.13 ± 0.27	1.00 ± 0.34	2.02 ± 1.19	3.88 ± 2.38	3.49 ± 0.71	5.01 ± 3.20

Values are mean ± SE from at least three different experiments relative to mRNA levels in AMs.

**Table 3:** Relative mRNA expression in AMs and lung fibroblast (fibro) cocultures stimulated with silica (Si; 200 μg/mL) for 4 hr.

*mRNA*	AM	AM + Si	Fibro	Fibro + Si	AM + Fibro	AM + Fibro + Si
*IL-1*β	1.03 ± 0.08	0.71 ± 0.12	0.08 ± 0.07	0.31 ± 0.29	1.49 ± 0.46	2.41 ± 0.91
*IL-10*	1.16 ± 0.11	1.04 ± 0.16	0.33 ± 0.17	0.78 ± 0.40	25.9 ± 15.8	26.8 ± 13.4
*iNOS*	1.14 ± 0.11	0.83 ± 0.19	0.58 ± 0.33	1.54 ± 0.93	8.22 ± 3.54	16.2 ± 9.95
*GM-CSF*	1.23 ± 0.21	1.06 ± 0.17	18.4 ± 7.0	58.9 ± 22.4	6.44 ± 2.4	58.0 ± 43.7

Values are mean ± SE from at least three different experiments relative to mRNA levels in AMs.

**Table 4:** Relative mRNA expression in AMs and lung fibroblasts (fibro) stimulated with silica (Si; 200 μg/mL) for 4 hr in Transwell experiments.

	Insert/well								
*mRNA*	AM*^a^*/none	None/fibro*^a^*	AM*^a^*/fibro	AM/fibro*^a^*	AM*^a^* + Si/fibro	AM + Si/fibro*^a^*	AM*^a^*/fibro + Si	AM*^a^*/fibro*^a^* + Si
*IL-1*β	1.0 ± 0.1	0.3 ± 0.2	4.7 ± 2.5	3.1 ± 2.5	1.1 ± 0.5	0.6 ± 0.2	2.8 ± 2.1	1.0 ± 0.5
*IL-6*	0.7 ± 0.1	912 ± 565	1.1 ± 0.8	14,487 ± 11,702	16 ± 9.6	3,009 ± 1,344	4.8 ± 4.3	14,573 ± 16,524
*IL-10*	1.4 ± 0.4	1.6 ± 0.6	1.2 ± 0.6	3.5 ± 2.0	0.8 ± 0.5	1.3 ± 1.1	0.1 ± 0.1	0.7 ± 0.4
*iNOS*	0.8 ± 0.1	1.0 ± 0.1	2.6 ± 1.4	3.4 ± 1.6	3.9 ± 2.8	1.9 ± 1.5	1.5 ± 1.1	1.0 ± 0.4
*GM-CSF*	1.0 ± 0.3	4.2 ± 1.9	2.1 ± 0.7	41.6 ± 24	1.6 ± 0.9	42 ± 39	0.9 ± 1.0	19.3 ± 7.2

Values are mean ± SE from three different experiments relative to mRNA levels in AMs.

a Source of the cells in which the mRNA levels were measured.
